# PLGA Membranes Functionalized with Gelatin through Biomimetic Mussel-Inspired Strategy

**DOI:** 10.3390/nano10112184

**Published:** 2020-11-02

**Authors:** Irene Carmagnola, Valeria Chiono, Gerardina Ruocco, Annachiara Scalzone, Piergiorgio Gentile, Paola Taddei, Gianluca Ciardelli

**Affiliations:** 1 Department of Mechanical and Aerospace Engineering, Politecnico di Torino, 10129 Torino, Italy; irene.carmagnola@polito.it (I.C.); gerardina.ruocco@polito.it (G.R.); gianluca.ciardelli@polito.it (G.C.); 2 POLITO BIOMedLAB, Politecnico di Torino, 10129 Turin, Italy; 3 Department for Materials and Devices of the National Research Council, Institute for the Chemical and Physical Processes (CNR-IPCF UOS), 56124 Pisa, Italy; 4 School of Engineering, Newcastle University, Claremont road, Newcastle upon Tyne NE1 7RU, UK; A.Scalzone2@newcastle.ac.uk (A.S.); Piergiorgio.Gentile@newcastle.ac.uk (P.G.); 5 Department of Biomedical and Neuromotor Sciences, University of Bologna, 40126 Bologna, Italy; paola.taddei@unibo.it

**Keywords:** PLGA, electrospinning, DOPA, biomimetic functionalization, surface modification

## Abstract

Electrospun membranes have been widely used as scaffolds for soft tissue engineering due to their extracellular matrix-like structure. A mussel-inspired coating approach based on 3,4-dihydroxy-DL-phenylalanine (DOPA) polymerization was proposed to graft gelatin (G) onto poly(lactic-co-glycolic) acid (PLGA) electrospun membranes. PolyDOPA coating allowed grafting of gelatin to PLGA fibers without affecting their bulk characteristics, such as molecular weight and thermal properties. PLGA electrospun membranes were dipped in a DOPA solution (2 mg/mL, Tris/HCl 10 mM, pH 8.5) for 7 h and then incubated in G solution (2 mg/mL, Tris/HCl 10 mM, pH 8.5) for 16 h. PLGA fibers had an average diameter of 1.37 ± 0.23 µm. Quartz crystal microbalance with dissipation technique (QCM-D) analysis was performed to monitor DOPA polymerization over time: after 7 h the amount of deposited polyDOPA was 71 ng/cm^2^. After polyDOPA surface functionalization, which was, also revealed by Raman spectroscopy, PLGA membranes maintained their fibrous morphology, however the fiber size and junction number increased. Successful functionalization with G was demonstrated by FTIR-ATR spectra, which showed the presence of G adsorption bands at 1653 cm^−1^ (Amide I) and 1544 cm^−1^ (Amide II) after G grafting, and by the Kaiser Test, which revealed a higher amount of amino groups for G functionalized membranes. Finally, the biocompatibility of the developed substrates and their ability to induce cell growth was assessed using Neonatal Normal Human Dermal Fibroblasts.

## 1. Introduction

Extracellular matrix (ECM) constitutes the natural environment interacting with cells: it is based on biopolymers arranged in a complex nanoscale topography and supplies haptotactic and chemotactic cues to cells. Electrospun matrices, characterized by ultrafine continuous fibers, high surface-to-volume ratio, high porosity, and variable pore-size distribution, can morphologically mimic the structure of the natural ECM and therefore are optimal substrates for Tissue Engineering (TE) [1,2]. Many synthetic and natural polymers have been electrospun to prepare fibrous membranes. Synthetic polymers offer easier processability and better mechanical properties, although their cell adhesion ability is generally lower [3].

On the contrary, natural polymers such as proteins (e.g., gelatin, collagen) and polysaccharides (e.g., chitosan) provide chemical cues which facilitate cell attachment [4].

Bioartificial materials are combinations of natural and synthetic polymers, aimed at synergistically combining their characteristics, to obtain new multicomponent materials with cell-adhesion ability and tailored mechanical properties and degradation rate [5,6,7,8]. Bioartificial tissue engineered scaffolds were prepared by electrospinning fibrous membranes in which the fiber core was made of a synthetic polymer and the shell consisted of a natural polymer guiding cell response [9]. Alternatively, bioartificial membranes were prepared by electrospinning blends of natural and synthetic polymers [10] or by grafting natural polymers onto electrospun membranes made of synthetic polymers [11].

Poly(lactic-co-glycolic) acid (PLGA) has been widely used as a material for biomedical applications due to its biocompatibility, tailored biodegradation rate, and mechanical properties [12].

Gelatin (G) is a natural polymer derived from collagen and it is used in different applications of tissue engineering and regenerative medicine. G is an interesting commercially available biomaterial because it is biodegradable, completely resorbable, does not express antigenicity in physiological conditions, and its physicochemical properties can be modulated by crosslinking [13,14].

Several studies reported the preparation of bioartificial mats obtained by electrospinning PLGA/gelatin blends [15,16]: the presence of gelatin decreased the diameter and the mechanical properties of membranes and increased their hydrophilicity with respect to PLGA scaffolds. Moreover, gelatin/PLGA membranes used as bone substitutes promoted the adhesion and proliferation of murine calvarial preosteoblasts (MC3T3-E1) compared to PLGA membranes.

In recent years, the design of nanoscale surface coatings mimicking the adhesive properties of marine mussels has gained great interest. The mechanism for mussel adhesion is mediated by byssal threads, which contain roughly 25–30 different proteins. All these proteins contain the post-translationally modified amino acid 3,4-dihydroxyphenyl-L-alanine (DOPA), which in its oxidized form (DOPAquinone) is thought to provide the moisture-resistance characteristic of mussel underwater adhesion [17]. DOPAquinone can self-polymerize to form thin polymeric films (polyDOPA) on virtually all types of materials. Simple immersion of substrates in an aqueous solution of DOPA or 3,4-(dihydroxyphenyl)ethylamine (dopamine) at a slightly alkaline pH (which is typical of marine environment) causes the spontaneous self-polymerization of the monomer and the deposition of a thin adherent polyDOPA or polydopamine film on the substrate [18,19]. Different polymeric electrospun membranes have been previously coated with polyDOPA or polydopamine with the aim to improve their cellular response. Rim et al. [20] fabricated electrospun poly(L-lactic) acid (PLLA) fibers modified by polydopamine coating as substrate to regulate adhesion, proliferation, and migration of human mesenchymal stem cells (hMSCs). The hMSC adhesion, alkaline phosphatase (ALP) activity and the expression of osteogenic genes were enhanced in the fibers coated with polydopamine, suggesting that mussel-inspired coating could regulate stem cell differentiation. Additionally, the behavior of human umbilical vein endothelial cells (HUVECs) was shown to be affected by polydopamine coating. Ku et al. [21] demonstrated that HUVECs, seeded on polycaprolactone (PCL) fibers coated with polydopamine, exhibited highly enhanced adhesion and viability compared to PCL substrates and also showed a positive expression of endothelial cell markers.

PolyDOPA and polydopamine coatings represent versatile strategies for grafting functional molecules on substrates, allowing their surface chemistry to be adapted to different applications. Particularly, mussel-inspired coatings react with a range of biomacromolecules, e.g., quinones (oxidized cathecols) and amino-containing molecules react via Michael addition or Schiff base reaction [22,23] and functional coatings may be obtained [20]. Yang et al. [24] exploited the ability of polyDOPA coating to easily graft adhesion peptides or neutrophic growth factors on different polymeric surfaces to improve differentiation and proliferation of human neuronal stem cells (NSCs). The grafting of growth factors or peptides via the mussel-inspired approach on polymeric substrates effectively improved differentiation and proliferation of human NSCs compared to Matrigel control.

Lee et al. [25] functionalized electrospun PLGA nanofibers with Bone Forming Peptide 1 (BFP1) using polydopamine as linker. BFP1-immobilised fibers enhanced the osteogenic differentiation of hMSCs compared to PLGA membranes. *In vivo* tests showed effective integration of bioactive nanofibers in mouse calvarial defects. Fibronectin was grafted onto polymeric fiber membranes by Xie and co-workers [26] in order to develop a system for multiple uses in tissue engineering. MIH3T3 cell culture demonstrated polydopamine coating improved adhesion and spreading and accelerated cytoskeletal maturation.

Based on the state of the art analysis, polydopamine or polyDOPA coatings have been widely employed for the surface grafting of biomolecules, although only a few studies have exploited the mussel-inspired approach to design bioartificial scaffolds for soft tissue engineering, such as skin [27], cartilage [18], and vascular [21] tissue. In the present study, PLGA fibrous membranes were developed through electrospinning, and surface grafted with gelatin exploiting a polyDOPA coating, with the final aim to obtain scaffolds for soft tissue engineering applications. The successful preparation of PLGA fibrous membranes was studied by Scanning Electron Microscopy (SEM). Attenuated Total Reflectance-Fourier Transform IR (ATR-FTIR), and Raman spectroscopies were used to characterize the samples at molecular scale, to assess the possible structural changes induced by the functionalization steps. ATR-FTIR spectra are representative of the sample surface, while Raman spectroscopy is more sensitive to sample bulk properties. Physicochemical characterizations were carried out to confirm the functionalization including QCM-D as a novel technique for the analysis of surface coatings. Finally, in vitro tests were performed using Neonatal Normal Human Dermal Fibroblasts (NHDF-Neo) in the perspective of future applications in the field of wound repair.

## 2. Materials and Methods

### 2.1. Materials

Poly(lactic-co-glycol) acid (LA:GA 75:25, Mw = 66–107 kDa, PLGA), 3,4-dihydroxy-DL-phenylalanine (DOPA) and type A gelatin from porcine skin (G) were purchased from Sigma-Aldrich (Milan, Italy). 2-Amino-2-(hydroxymethyl)-1,3-propanediol (TRIS), hydrochloric acid (HCl), and dichloromethane were purchased from Sigma-Aldrich (Milan, Italy).

All solvents were of analytical grade and used without further purification and supplied from Sigma-Aldrich.

### 2.2. Methods

#### 2.2.1. Fibrous Membrane Preparation

PLGA was dissolved in dichloromethane obtaining a solution with 22% w/v concentration. PLGA membranes were electrospun using a home-made electrospinning, equipped with a syringe pump (KD Scientific^®^ KDS100, Holliston, MA, USA). Electrospinning was performed at a feeding rate of 200 µL/min, an applied voltage of 15 kV, a distance between the collector and the 21-gauge needle of 20 cm and at 25 °C. Randomly oriented fibers were collected on a planar collector covered with aluminum paper. Membranes were obtained by electrospinning continuously about 4 mL of PLGA solution. All electrospun mats were dried in a fume hood overnight to eliminate any residual solvent. Final membranes had a thickness of ~0.2 µm measured by a caliper.

#### 2.2.2. Gelatin Functionalization of Fibrous Membranes Mediated by Mussel-Inspired Coating

DOPA solution (2 mg/mL) was prepared using Tris/HCl 10 mM at pH 8.5 as buffer solution. PLGA membranes were dipped in DOPA solution for 7 h at room temperature under continuous stirring (PLGA-DOPA). After this treatment, PLGA fibers were abundantly washed in Tris/HCl solution and then incubated in a G solution (2 mg/mL, Tris/HCl 10 mM, pH 8.5) for 16 h (PLGA-DOPA-G). Samples were finally washed with milliQ water and dried at room temperature. As control, PLGA membranes were coated with G by physical absorption (PLGA-G), by dipping PLGA membranes in G solution (2 mg/mL, Tris/HCl 10 mM, pH 8.5) for 16 h.

#### 2.2.3. QCM-D Analysis

The quartz crystal microbalance with dissipation technique (QCM-D) (Q-Sense Explorer, Biolin Scientific, Gothenburg, SE) was used to monitor DOPA-based coating formation. Gold coated quartz crystals (5 MHz) were used as model substrates and QCM-D was equipped with the open module to allow static condition analysis. Then 300 µL of DOPA solution (2 mg/mL) in Tris/HCl 10 mM at pH 8.5 was added in the open module for 7 h, recording continuously frequency and dissipation variation for multiple harmonics (up to the 13th overtone) as a function of time. Then three rinsing steps with bi-distilled water were performed to remove unreacted DOPA. The deposited mass on the crystal was calculated using the Sauerbrey equation, based on data collected for overtone *n* = 5.

#### 2.2.4. Scanning Electron Microscopy (SEM) analysis

Scanning electron microscopy was performed to evaluate the surface morphology of fibrous samples after each functionalization step. Before SEM analysis, samples were covered with a thin gold coating through a sputter coater (Agar Auto Sputter Coater, Agar Scientific, Essex, UK). Sample morphology was analyzed by using SEM LEO-1430 (Zeiss, Oberkochen DE), at two magnifications: 500× and 1000×. SEM images were processed through ImageJ software (National Institutes of Health, Bethesda, USA) to estimate the average value of fiber diameter, fiber diameter distribution, and pore size distribution.

#### 2.2.5. Static Contact Angle Analysis

The wettability of samples was evaluated through static contact angle analysis performed using the Attention Theta instrument (Biolin Scientific, Gothenburg, SE) and software OneAttension software (Biolin Scientific, Gothenburg, SE). Measures were carried out in static conditions on PLGA, PLGA-DOPA, PLGA-G, and PLGA-DOPA-G membranes and PLGA cast films using drops with 5 µL volume and collecting 3 images with a frame rate of 1 image per second. Measured data were averaged and standard deviation was calculated (*n* = 9).

#### 2.2.6. Infrared Analysis in ATR Modality (ATR-FTIR)

The ATR-FTIR technique was performed with the aim of detecting any modification of surface composition after each functionalization step. ATR-FTIR spectra were obtained through Perkin Elmer Spectrum One Spectrometer instrument (Perkin Elmer Italia, Milano, IT) was equipped with a Diamond crystal, at 4000–650 cm^−1^ wavenumber range, setting a resolution of 4 cm^−1^ and a scan number of 32. The spectra were analyzed by Spectrum software (Perkin Elmer Italia, Milano, IT).

#### 2.2.7. Differential Scanning Calorimetry (DSC)

Differential scanning calorimetry (DSC) was carried out in a DSC Q20 (TA INSTRUMENT, New Castle, UK) on samples (5–10 mg) packed in aluminum pans. The pan was heated from −20 °C to 200 °C at a heating rate of 10 °C min^−1^, isothermally maintained at 200°C for 3 min, cooled to −20 °C at 10 °C/min, and reheated from −20 to 200 C at 10 °C/min under nitrogen atmosphere. The glass transition temperatures (Tgs) of PLGA and PLGA-DOPA were measured from the second DSC heating scan. Analysis was carried out in triplicate for each sample type.

#### 2.2.8. Gel Permeation Chromatography (GPC)

Molecular weights (Mw, Mn) and molecular weight distributions (Mw/Mn) of samples were measured by gel permeation chromatography (GPC) using an Agilent system equipped with a Water 2414 RI detector (Agilent Technologies Italia, Leini, TO, IT). The measurements were performed using four Water columns (range 103–106 Å) at 40 °C; tetrahydrofuran (THF) as eluent (1.0 mL/min) and narrow polystyrene standards were used for calibration.

#### 2.2.9. Raman Spectroscopy

Raman spectra were recorded on a Bruker MultiRam FT-Raman spectrometer (Bruker Italia S.r.l., Milano, IT) equipped with a cooled Ge-diode detector. The excitation source was a Nd^3+^-YAG laser (1064 nm) in the backscattering (180°) configuration. The focused laser beam diameter was about 100 μm and the spectral resolution 4 cm^−1^. The spectra were recorded with laser power on the samples of about 60 mW without decomposition.

#### 2.2.10. Mechanical Tensile Tests

Changes in bulk mechanical properties after the functionalization process were evaluated on PLGA and PLGA-DOPA membranes through tensile tests using MTS QTest/10 (MTS Systems S.r.l., Torino, IT) equipped with Testwork 4 software ((MTS Systems S.r.l., Torino, IT). Samples were cut into “dog bone”-shapes and the sample thickness was measured by a calliper. A 10N load cell was used for the tests and a moving crosshead speed of 2 mm/min was set. At least three samples per scaffold type were tested. The stress–strain curves were plotted and the Young’s modulus E and maximum tensile strength (σ_max_) were calculated.

#### 2.2.11. Kaiser Test

The quantification of amino groups was carried out using the Kaiser test-kit purchased from Sigma-Aldrich (Milan, IT). Samples were cut in a square shape with area of about 0.5 cm^2^ and put in glass test tubes; 75 µL of phenol (~80% in ethanol), 100 µL of KCN (in H_2_O/ pyridine), and 75 µL of ninhydrin (6% in ethanol) were added subsequently to the samples and the test tubes were heated at 120°C for 5 min. Solutions were then diluted with 750 µL of H_2_O/ethanol, centrifugated and analyzed through Perkin Elmer^®^ Lambda 25 UV/Vis Systems (Perkin Elmer Italia, Milano, IT) at 450–700 nm. The concentration of amino groups (C) was calculated starting from the absorbance (A) value at 560 nm through the Lambert-Beer equation (A = C·ε·l), where ε is the extinction molar coefficient (15,000 mol^−^1 cm^−1^) and l is the optical length.

#### 2.2.12. Cell Culture and Seeding Protocol

Neonatal Normal Human Dermal Fibroblasts (NHDF-Neo) were purchased from Lonza Biosciences (Basel, CH) and cultured as recommended by the supplier. Briefly, cells were grown at 37 °C, 5% CO_2_, in Dulbecco’s Modified Eagle Medium (DMEM, Sigma Aldrich, Milano, IT) supplemented with 10% fetal bovine serum (FBS), 2 mM L-glutamine and 10% fetal bovine serum (FBS), and 100 U mL^−1^ penicillin/streptomycin (P/S). The PLGA and PLGA-DOPA-G electrospun membranes (15 mm diameter) were mounted in 24-multiwell cell crowns (Scaffdex, Tampere, FIN). Then, membranes were treated with Sudan Black (SB) to suppress autofluorescence. Each sample was covered with 50 µL of SB solution (0.3% w/v in ethanol), incubated for 30 min at 37 °C and washed properly twice with Dulbecco’s phosphate-buffered saline (DPBS). Before cell seeding, the membranes were sterilized under a UV lamp for 30 min. NHDF-Neo were suspended in DMEM and seeded on each sample at a concentration of 3 × 10^4^ cells per membrane and incubated at 37 °C, 5% CO_2_ for 30 min. Then, fresh DMEM was added up to 1 mL volume.

#### 2.2.13. Biological Tests

The cytocompatibility of the membranes was assessed using the Live/Dead assay (LIVE/DEAD^®^ Cell Imaging Kit, Life Technologies, UK), according to the manufacturer’s instructions. This fluorescence-based kit combines calcein AM and ethidium bromide to yield two-color discrimination of the population of live (green) from the dead (red) cells. Briefly, 4 μM ethidium homodimer−1 and 10 μM calcein were diluted in DPBS. Each sample was washed twice with DPBS before incubation with the staining solution for 30 min at 37 °C. Images were collected at day 1 and day 3 using a fluorescence microscope LEICA DM LB2 (Leica Microsystems Srl, Buccinasco, MI, IT).

Cells metabolic activity was analyzed with the Prestoblue assay up to 8 days. Briefly, cell culture medium was removed at each time point (1, 4, and 8 days), and samples were washed with DPBS. Then 1 mL of PrestoBlue™ reagent (Thermo Scientific, UK) diluted in DMEM (1:10) and protected from light was added to each membrane and incubated for 2 h at 37 °C and 5% CO_2_. Then, 200 µL of each well solution was transferred in triplicate to a white bottom 96-well plate and an LS−50B Luminescence Spectrometer (Perkin Elmer, Waltham, MA, USA) was used to measure the fluorescence (at excitation/emission of 560/590 nm). Then, samples were washed twice with DPBS and fresh medium was added for the next time point.

The immunostaining analysis was performed fixing the cellularized samples in pre-warmed 4% w/v paraformaldehyde (PFA) for 15 min and cells were consequently permeabilised using 0.1% v/v Tween20^®^ (Life Science, Sigma-Aldrich, UK) in DPBS for three washes. The cells cytoskeleton was stained using Rhodamine-phalloidin, prepared using phalloidin-tetramethylrhodamine B isothiocyanate (1:100 in 0.1% DPBS/Tween20^®^) for 20 min at room temperature. Then, for staining the cells nuclei samples were washed with 0.1% DPBS/Tween20^®^ solution and immersed in 4′,6-diamidino-2-phenylindole (DAPI) solution (Vector Laboratories, Peterborough, UK) (1:2500 in 0.1% DPBS/Tween20^®^) for 10 min at room temperature. At day 3, images were collected using a Nikon A1R inverted confocal microscope.

#### 2.2.14. Statistical Analysis

Experiments were repeated three times and results expressed as a mean ± standard deviation. Statistical significance was calculated using analysis of variance (ANOVA). A comparison between two means was analyzed using Tukey’s test with statistical significance level set at *p < 0.05 and. **p < 0.001.

## 3. Results

### 3.1. Characterization of the Polydopa Coating

In this work, electrospun PLGA membranes were prepared and surface grafted with G through a polyDOPA intermediate coating, following a mussel inspired approach.

**Figure 1:**
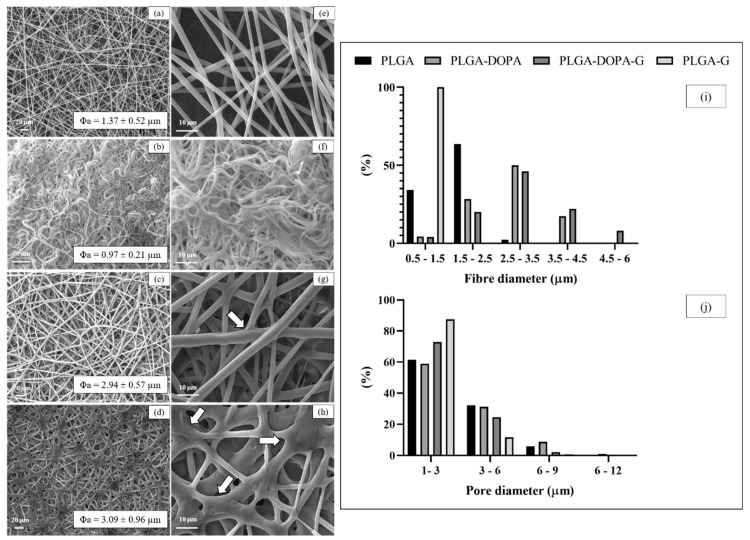
SEM images of the electrospun PLGA (**a**,**e**); PLGA-G (**b**,**f**); PLGA-DOPA (**c**,**g**); PLGA-DOPA-G (**d**,**h**) fibers. Average value of fiber diameters (**a**–**d**). Fiber diameter (**i**) and pore diameter distribution (**j**). White arrows indicate fiber junction.

The morphology, the average diameter of fibrous membranes, and pore and diameter distribution were analyzed by SEM (Figure 1). PLGA and PLGA-DOPA fibrous membranes were randomly oriented, and presented a porous network without defects, which is expected to support cell adhesion, and to be permeable to metabolites and catabolites. The average diameter of PLGA and PLGA-DOPA fibers was 1.37 ± 0.52 µm and 2.94 ± 0.57 µm, respectively. After polyDOPA deposition, the average fiber diameter significantly increased, (Figure 1b,e,i,j) [28], while pore size distribution was not significantly affected by the presence of the polyDOPA coating.

**Figure 2:**
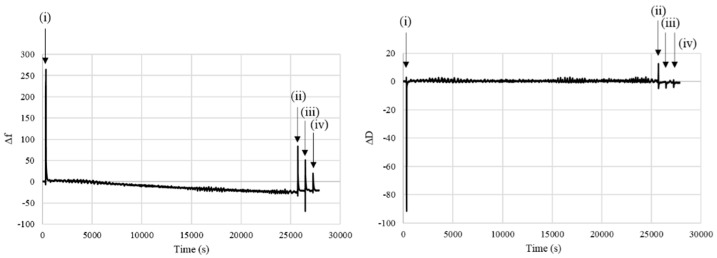
The Δ*f* and Δ*D* signals (5th overtones) over time of DOPA deposition on gold substrates; four events were identified: (i) DOPA solution insertion and (ii), (iii), and (iv) washing steps.

The growth of the polyDOPA coating was followed through QCM-D analysis (Figure 2), moreover this technique was employed to establish the coating characteristics (rigid or soft/viscoelastic behavior) and to estimate the amount of deposited polyDOPA and the coating thickness The dissipation value did not significantly change during polyDOPA polymerization suggesting that the polyDOPA coating formed a rigid layer. Consequently, the frequency shift (Δ*f*) was proportional to the mass change after deposition. The mass change could be quantified by the Sauerbrey equation [29]: after the washing steps, the amount of polyDOPA deposited on the gold substrates was 71 ng/cm^2^ with an estimated thickness of ~4 nm.

**Figure 3:**
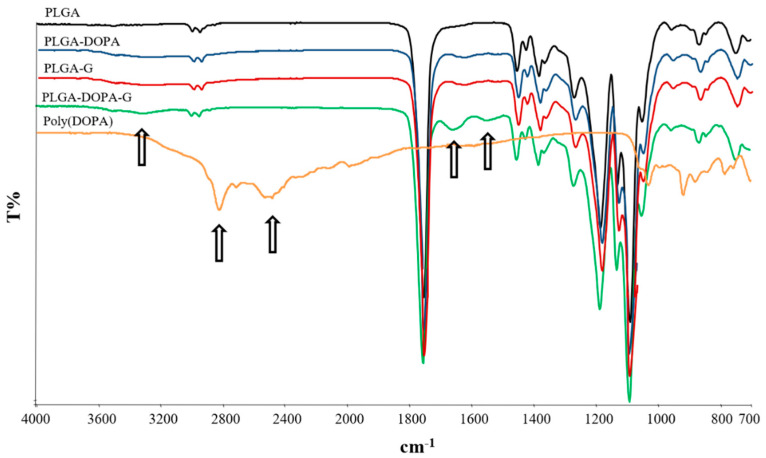
ATR-FTIR spectra of electrospun PLGA (black), PLGA-DOPA (blue), PLGA-G (red) and PLGA-DOPA-G (green) fibers and poly(DOPA) film (orange).

Infrared and Raman spectroscopy were used to further characterize the formation of poly(DOPA) onto PLGA membranes. Figure 3 reports the ATR-FTIR spectra of PLGA and PLGA-DOPA membranes and a poly(DOPA) film. The PLGA ATR-FTIR spectrum showed the absorption bands at 1750 and 1080 cm^−1^ attributable to carbonyl and C–O–C groups, respectively, confirming the presence of the ester and ether groups. The ATR-FTIR spectrum of the poly(DOPA) film showed the characteristic absorption bands reported in the literature [19,30,31].

With respect to the PLGA ATR-FTIR spectrum, the PLGA-DOPA spectrum showed the presence of new absorption bands attributable to polyDOPA deposition: the wide absorption band at 3600–3000 cm^−1^ was due to stretching vibrations of –NH and –OH functional groups of polyDOPA, the band at 1631 cm^−1^ was attributed to C=C vibration of polyDOPA aromatic rings, whereas the band at 1526 cm^−1^ was due to N–H bending vibrations.

**Figure 4:**
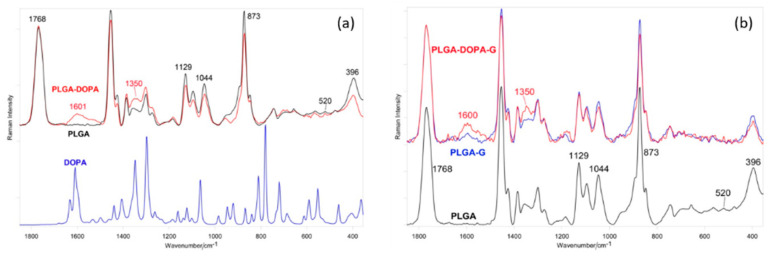
Raman spectra of (**a**) DOPA powder, PLGA and PLGA-DOPA fibers; (**b**) PLGA, PLGA-G and PLGA-DOPA-G fibers.

The Raman spectrum of PLGA (Figure 4) was dominated by the bands of lactic acid units, as expected on the basis of the 75:25 LA:GA ratio; the polymer structure appeared unordered, as revealed by the absence of any prominent band at 924 cm^−1^, identified as a marker of the 10_3_ helical α-crystal structure of poly(lactic) acid (PLA) [32].

The spectrum below 600 cm^−1^ confirmed the absence of crystallinity, which is typical of PLGA. The band at 520 cm^−1^ (due to the crystalline form of PLA) was detected with a very low intensity, and a broad single CCO bending component at 396 cm^−1^ was observed instead of the well-defined doublet at 410–397 cm^−1^, typical of a PLA crystalline polymer [28,33]. No prominent components at 205 and 158 cm^−1^ (due to skeletal modes in crystalline PLA) were detected.

Upon PLGA coating with polyDOPA, the Raman spectrum of PLGA-DOPA showed an increase of band intensity at 1600 and 1350 cm^−1^, due to the aromatic groups of polyDOPA [20,30,34]. The structure of the PLGA fibrous membranes did not change after polyDOPA coating. In more detail, the spectral profile and width of the C=O stretching band at 1768 cm^−1^, and the I_1129_/I_1044_ and A_396_/A_873_ intensity ratios did not vary after functionalization. The former ratio (calculated as peak height) has proved sensitive to changes in conformational distribution (and thus crystallinity) [35], the latter (calculated as peak area) has been reported to increase with PLA crystallinity [33].

**Figure 5:**
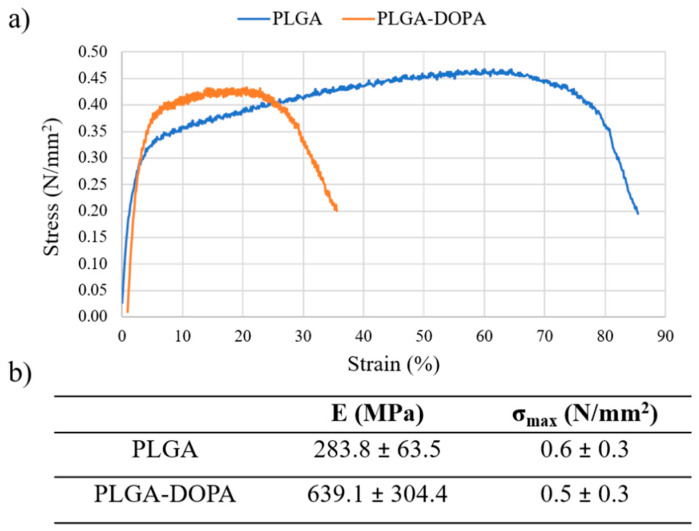
(**a**) Stress-strain curve obtained by testing PLGA and PLGA-DOPA membranes; (**b**) Young’s modulus (***E***), maximum tensile stress (***σ_Rmax_***) reported as average value ± standard deviation (*n* = 3).

Tensile test results, reported in Figure 5, demonstrated that the functionalization process did not affect the bulk properties of PLGA membranes: Young’s modulus and maximum tensile stress did not significantly change for PLGA-DOPA membranes (E = 639.1 ± 304.4 MPa; σ_max_ = 0.5 ± 0.3 N/mm^2^) compared to PLGA nanofibers (E = 283.8 ± 63.5 MPa; σmax = 0.6 ± 0.3 N/mm^2^).

**Figure 6:**
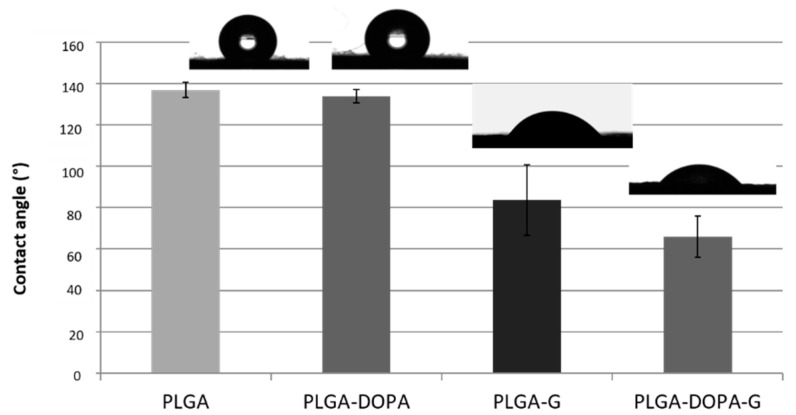
Static contact angle values of electrospun PLGA, PLGA-DOPA, PLGA-G and PLGA-DOPA-G membranes. Static contact angle was expressed as mean ± standard deviation.

PLGA film wettability increased after polyDOPA polymerization from 80° ± 0.5° to 61° ± 8° in agreement with that reported by Ku et al. [21], while the contact angle value of polyDOPA film was 34° ± 6°. Variations in surface wettability of electrospun membranes are reported in Figure 6. PLGA fibrous membranes showed a high contact angle value of 137 °± 4° and no significant variations were observed after polyDOPA film deposition (135° ± 3°). The higher contact angle values of porous fibrous membranes with respect to compact films were attributed to the porous structure of the membranes.

During incubation of PLGA membranes in DOPA solution, PLGA could undergo degradation phenomena causing a decrease in its molecular weight and Tg value, in turn affecting the membrane mechanical properties and stability in physiological conditions. For this reason, the chemical and thermal properties of PLGA-based membranes were evaluated before and after DOPA treatment by GPC and DSC analyses respectively, and results are collated in Table 1. Tg values of PLGA-DOPA and untreated PLGA membranes were similar: 48.9 ± °C and 49.2 ± °C, respectively. Similarly, the molecular weight (Mn, Mw, Mz, and polydipersity index - PDI) of PLGA samples did not change after DOPA treatment. These results demonstrated that bulk properties of membranes were not affected by the surface treatment.

### 3.2. Characterization of Membrane Functionalization with Gelatin

SEM images reported in Figure 1c,g demonstrated that, after G functionalization (PLGA-DOPA-G), the fibrous structure of the membranes was preserved, while the fiber diameter increased (3.09 ± 0.96 μm) compared to PLGA-DOPA and PLGA samples, and the pore size slightly decreased (Figure 1j). Additionally, the number of fiber junctions in PLGA-DOPA-G membranes increased with respect to PLGA-DOPA samples. On the contrary, control PLGA-G fibrous membranes only showed a slight increase of the average fiber diameter (0.971 ± 0.21 μm) with respect to the non-treated fibrous membranes. SEM images of PLGA-G membranes (Figure 1b,f) did not show the formation of new junctions among the fibers. Furthermore, fibers in PLGA-G membranes evidenced a curly geometry which could be attributed to PLGA fibers relaxation during the incubation time in the G solution. Such geometry was not observed when polyDOPA coating was present on the fibers suggesting the additional ability of polyDOPA coating in stabilizing the fiber geometry.

ATR-FTIR spectra of PLGA-G and PLGA-DOPA-G fibrous membranes are reported in Figure 3 and are compared to the spectra of PLGA and PLGA-DOPA samples. After G functionalization, the ATR-FTIR spectrum of PLGA membranes pretreated with DOPA showed the characteristic absorption bands of G [13,36]: amide I and amide II bands at 1653 and 1544 cm^−1^ respectively, and a broad band centered at 3300 cm^−1^ attributed to the N–H and O–H stretching vibrations. The ATR-FTIR spectrum of PLGA-G fibrous membranes showed the same absorption bands of the PLGA-DOPA-G spectrum, although at a significantly lower intensity.

The Raman spectrum of PLGA-G fibrous membranes (Figure 4) was dominated by the bands of the PLA component. Again, Raman spectroscopy evidenced the well-known amorphous structure of PLGA, as revealed by the absence of any prominent band at 924 cm^−1^, identified as a marker of the 10_3_ helical α-crystal structure of PLA [32]. Moreover, in PLGA-G fibrous membranes, no detectable bands ascribable to the G component were observed. Instead, the PLGA-DOPA-G spectrum showed a significant increase of signal near 1600 and 1350 cm^−1^, due to the aromatic groups of polyDOPA, as observed in PLGA-DOPA samples.

After gelatin functionalization, the surface wettability of membranes increased significantly compared to untreated (137° ± 4°) and polyDOPA treated (134° ± 3°) samples (Figure 6): the static contact angle value measured for PLGA-G fibrous membranes was 84° ± 17°, which was further reduced to 66° ± 10° for PLGA-DOPA-G.

The Kaiser test was performed to evaluate the amino group density on the membranes (Table 2). The test principle is the reaction of ninhydrin with a primary amine, having a hydrogen atom bound to the C_α_, to form Ruheman’s blue, which has a highly conjugated structure responsible for a strong absorption at 570 nm. The amino group density was higher for the PLGA-DOPA-G membranes compared to PLGA and PLGA-DOPA samples (58.9 ± 5.8 nmol/cm^2^, 2.49 ± 0.26 nmol/cm^2^ and 29.2 ± 11.6 nmol/cm^2^, respectively).

### 3.3. Cell Tests

**Figure 7:**
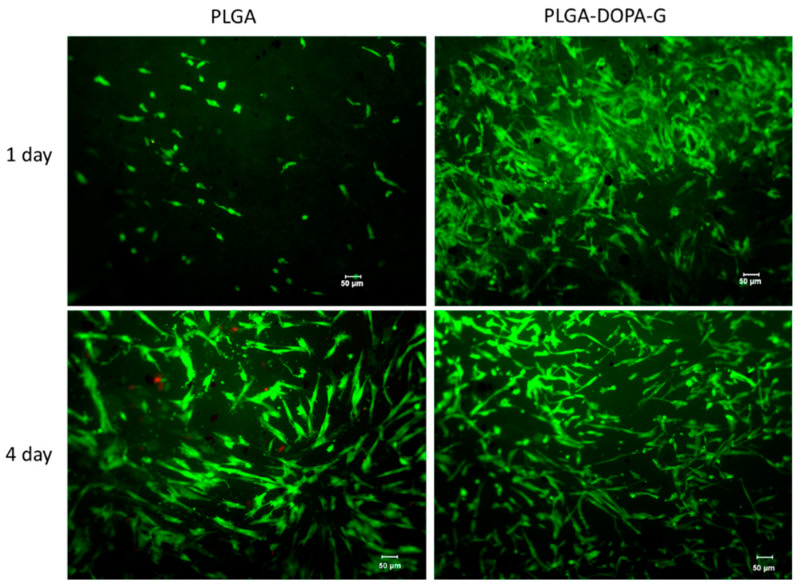
Live/Dead assay of cells seeded on membrane surfaces. Representative fluorescent micrographs of live (green) and dead (red) cells seeded on PLGA and PLGA-DOPA-G based fibers after 1 and 4 days. Scale bars = 50 µm.

**Figure 8:**
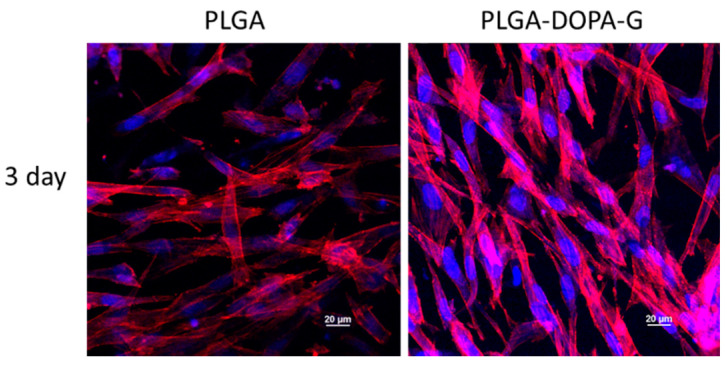
Immunostaining analysis of cells seeded on membrane surfaces. Representative fluorescent micrographs of cell morphology (nucleus stained with 4′,6-diamidino-2-phenylindole (DAPI) in blue, and actin filaments stained with Rhodamine in red) on PLGA and PLGA-DOPA-G based fibers after 3 days culture time. Scale bars = 20 µm.

**Figure 9:**
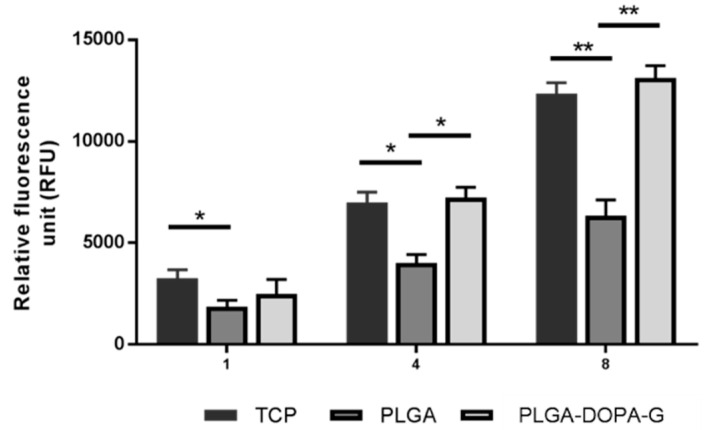
PrestoBlue assay of cells cultured on based fibrous PLGA and PLGA-DOPA-G membranes after 1, 4, and 8 days (* *p* < 0.05 and ** *p* < 0.01). TCP (control): tissue culture plates.

Cell viability on PLGA and PLGA-DOPA-G samples was evaluated by live/dead staining assay after 1 and 3 days of incubation, as shown in Figure 7. The PLGA-DOPA-G membranes showed a higher cell viability and ability to stimulate cell attachment compared to the PLGA membrane at day 1. However, cell viability on PLGA membrane increased after 3 days culture time showing a reduction of dead cells. In terms of cell morphology, NHDF-Neo exhibited their typically flattened and elongated shape on both types of membranes at 3 days and spread homogeneously along the sample surface as shown by immunostaining assays (Figure 8). The metabolic activity of NHDF-Neo was evaluated using Presto Blue assay up to 8 days (Figure 9). NHDF-Neo metabolic activity increased from day 1 to day 8 in both samples. Interestingly, cells on functionalized PLGA membranes showed a significantly higher metabolic activity compared to PLGA membranes (Relative fluorescence unit: 14,128 ± 158 and 7236 ± 88 for PLGA-DOPA-G and PLGA respectively at 8 days), suggesting a positive effect of a biomimetic environment on cell behavior.

## 4. Discussion

Scaffolds for soft tissue engineering were prepared based on electrospun PLGA membranes surface grafted with gelatin through polyDOPA intermediate coating.

PLGA has been extensively investigated for temporary medical applications, such as the design of controlled drug/protein delivery systems and scaffolds for tissue engineering [37,38]. Electrospun matrices show morphological similarities to the natural ECM, characterized by ultrafine continuous fibers, high surface-to-volume ratio, and high porosity. In this work, electrospun PLGA membranes consisting of microfibers with an average diameter of 1.37 ± 0.52 µm were prepared. The static contact angle of electrospun PLGA membranes (137° ± 4°) was significantly higher than for PLGA cast films (80° ± 0.5°) as displayed in Figure 5. Surface material properties depend on surface chemistry and micro- and nanoarchitecture: surface micro-structuring of electrospun PLGA fibrous membranes, particularly the presence of micropores, contributing to increase the material hydrophobicity (Figure 2). This result was in agreement with that reported by Park at al. who described the strong hydrophobicity feature of the PLGA nanofibers (~134°). [39]

PLGA is biocompatible but does not possess ligands for binding specific cell integrins; additionally, the surface hydrophobicity of electrospun PLGA membranes negatively affects cell attachment. Figure 6 and Figure 8 show that cells poorly adhered on electrospun PLGA membranes with respect to control tissue culture plates (TCP): cell number on PLGA membranes was around 50% with respect to TCP after 8 days, although it slightly increased with increasing time, evidencing cell proliferation.

For this reason, PLGA membranes were surface modified by grafting gelatin as an adhesion protein. Due to the lack of lateral reactive functional groups along the PLGA backbone, electrospun PLGA membranes were initially surface modified through an approach inspired by the adhesion mechanism of mussels. Mussels adhesion is mediated by specific proteins present in the attachment plaques of byssal threads, which contain a significant amount of DOPA aminoacid. At alkaline pH and oxidative conditions, DOPA converts itself into reactive DOPAquinone: if a substrate is immersed in a DOPA solution at pH 8.5 in aerated conditions, DOPAquinone forms and self- polymerizes, partially depositing on the substrate as a thin polyDOPA layer. The main features of a mussel-inspired pre-modification treatment with poyDOPA are its simplicity, low cost, as well as versatility: the polyDOPA layer may virtually adhere to any type of organic or inorganic material [40]. Furthermore, under oxidative conditions, organic molecules, such as proteins, may be grafted onto the polyDOPA coating: under oxidizing conditions, cathecol groups on the polyDOPA coating are converted into quinone groups, which then react with molecules containing thiol or amino groups [41]. In this work a mussel-inspired polyDOPA coating was employed as a linker-layer to graft gelatin onto PLGA fibrous membranes.

QCM-D analysis was applied for the first time to monitor real-time polyDOPA coating formation by measuring changes in frequency and energy dissipation of the system composed of a thin polyDOPA film adsorbed on a piezoelectric quartz sensor [42]. PolyDOPA formed a rigid film on a gold coated sensor, as demonstrated by ΔD = 0. Consequently, the measured frequency shift (Δ*f*) was proportional to the mass change and this could be quantified by the Sauerbrey equation [29]. Based on QCM-D analysis, the polyDOPA coating density resulted in being 71 ng/cm^2^.

Poly(DOPA) coating deposition was also confirmed by SEM: beside causing an increment of fiber average diameter, polyDOPA coating also formed some nano-agglomerates on the fiber surface, in agreement with that reported by Rim and co-workers [20]. ATR-FTIR and Raman spectroscopy were used to evaluate at a molecular scale possible modifications of the surface chemistry after each functionalization step. ATR-FTIR spectrum of PLGA-DOPA membranes showed the co-presence of the characteristic absorption bands of PLGA and polyDOPA, suggesting successful DOPA polymerization on the PLGA membrane [28]. Similarly, Raman spectroscopy confirmed the presence of polyDOPA coating through the appearance of additional peaks at 1600 and 1350 cm^−1^ (due to the aromatic groups in polyDOPA), compared to the PLGA spectrum [21]. The static contact angle of cast films decreased from 80°± 0.5° for the PLGA film to 61.0°± 8.0° for the PLGA-DOPA film. This result suggested that polyDOPA deposition was successful, although the high standard deviation for contact angle values measured could be attributed mainly to the impossibility to precisely control the membrane microstructure through the electrospinning techniques, since the contact angle value depends both on the chemical surface and the surface morphology (roughness). The surface hydrophobicity of the fibrous membrane did not change after the deposition of polyDOPA coating (137° ± 4° versus 134° ± 3° for PLGA-DOPA), probably due to the non-homogeneous polyDOPA coating and the surface microstructure of the membrane [43,44,45].

PolyDOPA coating was obtained in aqueous media; therefore this process could potentially cause PLGA partial degradation by hydrolysis. However, DSC and GPC results showed that thermal properties and molecular weight of PLGA-based samples (Table 1) did not vary after polyDOPA deposition. Further confirming DSC and GPC findings, tensile tests showed that mechanical properties were not influenced by the polyDOPA functionalization process.

Gelatin is obtained by thermal denaturation or physical and chemical degradation of collagen, the most widespread protein in the body occurring in most connective tissues as skin, tendons and bone. With respect to collagen, gelatin does not express antigenicity in physiological conditions [46]. In this work gelatin functionalization was applied to improve the biocompatibility and cell adhesion properties of PLGA membranes. Gelatin was selected as a biomimetic low cost material for surface functionalization. After gelatin grafting (mediated by polyDOPA coating) the fiber diameter and the number of fiber junctions increased compared to the samples functionalized with gelatin by simple physical adsorption (Figure 1b,d, respectively). These results suggested that the amount of immobilized gelatin was probably higher on PLGA-DOPA-G membranes as compared to PLGA- G membranes. This hypothesis was confirmed by further results: ATR-FTIR analysis (Figure 3) showed that the characteristic gelatin absorption bands were more intense in the PLGA-DOPA-G spectrum with respect to the PLGA-G spectrum. Additionally, although gelatin adsorption (PLGA-G membranes) caused a decrease in the static contact angle value (84° ± 17°), as shown in Figure 5, a still lower contact angle value was measured for PLGA-DOPA-G membranes (66° ± 10°). The high standard deviation values were probably attributable to non-homogeneous G coating on microstructured membranes. Finally, quantification of G on the sample surface was performed through the Kaiser test (Table 2), demonstrating a higher amount of gelatin in the presence of polyDOPA coating with respect to simple G physical adsorption: 58.9 ± 5.8 and 26.4 ± 3.3 nmol/cm^2^ respectively (Table 1).

The Raman spectra of PLGA-G and PLGA-DOPA-G did not show any band due to gelatin, while ATR-FTIR spectroscopy detected G; on the other hand, Raman spectroscopy was able to reveal polyDOPA. This result does not appear unexpected since ATR-FTIR spectra are representative of the surface chemical features of the specimens, while Raman spectroscopy is more sensitive to sample bulk chemistry. Moreover, the Raman spectrum of polyDOPA is intrinsically stronger than that of gelatin.

PolyDOPA coating and gelatin grafting were performed in aqueous media; therefore these processes could potentially cause PLGA partial degradation by hydrolysis. However, DSC and GPC results showed that the thermal properties and molecular weight of PLGA-based samples (Table 1) did not vary after polyDOPA and G functionalization steps.

Cytocompatibility tests were performed using NHDF-Neo cells in order to analyze the effect of the biomimetic functionalization on cell viability and morphology. Gelatin-grafted membranes via polyDOPA strongly supported cellular adhesion, due to the presence of cell-binding motifs (RGD) in gelatin, which can mediate cell-substrate interactions, enhancing cell proliferation as demonstrated by the Live and Dead results (Figure 7). Additionally, gelatin grafting decreased the surface contact angle value of PLGA membranes, which is beneficial for cell adhesion (Figure 6). Similar results have been reported in the literature [11,47], when gelatin was physically adsorbed or grafted via carbodiimide mediated reaction on PCL and PLLA membranes: functionalization improved endothelial cell spreading and proliferation.

PLGA-DOPA-G also improved cellular response compared to PLGA membranes as confirmed by the spreading morphology of fibroblasts (Figure 8) and enhanced cell metabolic activities (Figure 9). Indeed, metabolic activity on PLGA-DOPA-G fibrous membranes was similar compared to tissue culture plates control samples after 8 days.

## 5. Conclusions

In conclusion, in this work PLGA-DOPA-G membranes were prepared through a simple and versatile approach, as novel biomimetic bioartificial substrates, and their proof-of-principle use for soft tissue engineering was demonstrated by preliminary *in vitro* cell tests.

The mussel-inspired method used to graft G on electrospun PLGA membranes was simple, of low cost, employed mild conditions, preserved the morphological features, did not alter the bulk properties of the membranes, and was highly versatile.

The functionalization approach was characterized at each step, and interestingly QCM-D analysis was applied for the first time to analyze polyDOPA successful deposition. Preliminary *in vitro* cell tests confirmed that biomimetic functionalization of PLGA electrospun membranes strongly supported cellular adhesion and viability as compared to unfunctionalized membranes and positive control (TCP). This result was due to the presence of cell-binding peptides and surface hydrophilicity after gelatin grafting.

In the future, the same versatile functionalization approach could be easily applied to different substrates for different final applications.

## Figures and Tables

**Table 1:** Molecular weight and thermal property of PLGA and PLGA-DOPA treatment.

	Tg (°C)	Mn (g/mol)	Mw (g/mol)	Mz (g/mol)	Polydispersity Index (PDI)
**PLGA**	48.9	40,700	68,600	96,600	1.69
**PLGA-DOPA**	49.2	46,900	72,000	98,500	1.54

**Table 2:** Amino group density as evaluated by Kaiser-test on PLGA, PLGA-DOPA, PLGA-G and PLGA-DOPA-G samples.

	Abs	mol/L (×10^−5^)	nmol/cm^2^
**PLGA**	0.05 ± 0.01	4.97 ± 0.53	2.49 ± 0.26
**PLGA-DOPA**	0.06 ± 0.02	5.84 ± 2.32	29.2 ± 11.60
**PLGA-G**	0.05 ± 0.01	5.28 ± 0.65	26.4 ± 3.27
**PLGA-DOPA-G**	0.12 ± 0.01	11.8 ± 1.16	58.9 ± 5.77

## References

[1] Wang X., Drew C., Lee S.-H., Senecal K.J., Kumar J., Samuelson L.A. (2002). Electrospun Nanofibrous Membranes for Highly Sensitive Optical Sensors. Nano Lett..

[2] Agarwal S., Wendorff J.H., Greiner A. (2008). Use of electrospinning technique for biomedical applications. Polymer.

[3] Yoo H.S., Kim T.G., Park T.G. (2009). Surface-functionalized electrospun nanofibers for tissue engineering and drug delivery. Adv. Drug Deliv. Rev..

[4] Tonda-Turo C., Cipriani E., Gnavi S., Chiono V., Mattu C., Gentile P., Perroteau I., Zanetti M., Ciardelli G. (2013). Crosslinked gelatin nanofibres: Preparation, characterisation and in vitro studies using glial-like cells. Mater. Sci. Eng. C.

[5] Boffito M., Di Meglio F., Mozetic P., Giannitelli S.M., Carmagnola I., Castaldo C., Nurzynska D., Sacco A.M., Miraglia R., Montagnani S. (2018). Surface functionalization of polyurethane scaffolds mimicking the myocardial microenvironment to support cardiac primitive cells. PLoS ONE.

[6] Ciardelli G., Chiono V., Vozzi G., Pracella M., Ahluwalia A., Barbani N., Cristallini A.C., Giusti† P. (2005). Blends of Poly-(ε-caprolactone) and Polysaccharides in Tissue Engineering Applications. Biomacromolecules.

[7] Chiono V., Vozzi G., D’Acunto M., Brinzi S., Domenici C., Vozzi F., Ahluwalia A., Barbani N., Giusti P., Ciardelli G. (2009). Characterisation of blends between poly(ε-caprolactone) and polysaccharides for tissue engineering applications. Mater. Sci. Eng. C.

[8] Chiono V., Ciardelli G., Vozzi G., Cortez J., Barbani N., Gentile P., Giusti P. (2008). Enzymatically-Modified Melt-Extruded Guides for Peripheral Nerve Repair. Eng. Life Sci..

[9] Schnell E., Klinkhammer K., Balzer S., Brook G., Klee D., Dalton P., Mey J. (2007). Guidance of glial cell migration and axonal growth on electrospun nanofibers of poly-ε-caprolactone and a collagen/poly-ε-caprolactone blend. Biomaterials.

[10] Chong E.J., Phan T.T., Lim I.J., Zhang Y.Z., Bay B.H., Ramakrishna S., Lim C.T. (2007). Evaluation of electrospun PCL/gelatin nanofibrous scaffold for wound healing and layered dermal reconstitution. Acta Biomater..

[11] Ma Z., He W., Yong T., Ramakrishna S. (2005). Grafting of Gelatin on Electrospun Poly(caprolactone) Nanofibers to Improve Endothelial Cell Spreading and Proliferation and to Control Cell Orientation. Tissue Eng..

[12] Gentile P., Chiono V., Carmagnola I., Hatton P.V. (2014). An Overview of Poly(lactic-co-glycolic) Acid (PLGA)-Based Biomaterials for Bone Tissue Engineering. Int. J. Mol. Sci..

[13] Chiono V., Carmagnola I., Gentile P., Boccafoschi F., Tonda-Turo C., Ballarini M., Georgieva V., Georgiev G., Ciardelli G. (2012). Layer-by-layer coating of photoactive polymers for biomedical applications. Surf. Coatings Technol..

[14] Gentile P., Chiono V., Boccafoschi F., Baino F., Vitale-Brovarone C., Vernè E., Barbani N., Ciardelli G. (2010). Composite Films of Gelatin and Hydroxyapatite/Bioactive Glass for Tissue-Engineering Applications. J. Biomater. Sci. Polym. Ed..

[15] Meng Z., Wang Y., Ma C., Zheng W., Li L., Zheng Y. (2010). Electrospinning of PLGA/gelatin randomly-oriented and aligned nanofibers as potential scaffold in tissue engineering. Mater. Sci. Eng. C.

[16] Meng Z., Xu X., Zheng W., Zhou H., Li L., Zheng Y., Lou X. (2011). Preparation and characterization of electrospun PLGA/gelatin nanofibers as a potential drug delivery system. Colloids Surfaces B Biointerfaces.

[17] Silverman H.G., Roberto F.F. (2007). Understanding Marine Mussel Adhesion. Mar. Biotechnol..

[18] Tsai W.-B., Chen W.-T., Chien H.-W., Kuo W.-H., Wang M.-J. (2011). Poly(dopamine) coating of scaffolds for articular cartilage tissue engineering. Acta Biomater..

[19] Cheng C., Li S., Zhao W., Wei Q., Nie S., Sun S., Zhao C. (2012). The hydrodynamic permeability and surface property of polyethersulfone ultrafiltration membranes with mussel-inspired polydopamine coatings. J. Membr. Sci..

[20] Rim N.G., Kim S.J., Shin Y.M., Jun I., Lim N.W., Park J.H., Shin H. (2012). Mussel-inspired surface modification of poly(l-lactide) electrospun fibers for modulation of osteogenic differentiation of human mesenchymal stem cells. Colloids Surfaces B Biointerfaces.

[21] Ku S.H., Park C.B. (2010). Human endothelial cell growth on mussel-inspired nanofiber scaffold for vascular tissue engineering. Biomaterials.

[22] Burzio L.A., Waite J.H. (2000). Cross-Linking in Adhesive Quinoproteins: Studies with Model Decapeptides. Biochemistry.

[23] Lavoie M.J., Ostaszewski B.L., Weihofen A., Schlossmacher M.G., Selkoe D.J. (2005). Dopamine covalently modifies and functionally inactivates parkin. Nat. Med..

[24] Yang K., Lee J.S., Kim J., Bin Lee Y., Shin H., Um S.H., Kim J.B., Park K.I., Lee H., Cho S.-W. (2012). Polydopamine-mediated surface modification of scaffold materials for human neural stem cell engineering. Biomaterials.

[25] Lee Y.J., Lee J.-H., Cho H.-J., Kim H.K., Yoon T.-R., Shin H. (2013). Electrospun fibers immobilized with bone forming peptide-1 derived from BMP7 for guided bone regeneration. Biomaterials.

[26] Xie J., Michael P.L., Zhong S., Ma B., MacEwan M.R., Lim C.T. (2012). Mussel inspired protein-mediated surface modification to electrospun fibers and their potential biomedical applications. J. Biomed. Mater. Res. Part A.

[27] Zhao J., Han F., Zhang W., Yang Y., You D., Li L. (2019). Toward improved wound dressings: Effects of polydopamine-decorated poly(lactic-co-glycolic acid) electrospinning incorporating basic fibroblast growth factor and ponericin G1. RSC Adv..

[28] Nardo T., Chiono V., Ciardelli G., Tabrizian M. (2015). PolyDOPA Mussel-Inspired Coating as a Means for Hydroxyapatite Entrapment on Polytetrafluoroethylene Surface for Application in Periodontal Diseases. Macromol. Biosci..

[29] Sauerbrey G. (1959). Verwendung von Schwingquarzen zur Wäigung diinner Schichten und zur Mikrowäigung (Use of quartz crystals for weighing thin layers and for microweighing). Z. Phys..

[30] Xi Z.-Y., Xu Y.-Y., Zhu L.-P., Wang Y., Zhu B.-K. (2009). A facile method of surface modification for hydrophobic polymer membranes based on the adhesive behavior of poly(DOPA) and poly(dopamine). J. Membr. Sci..

[31] Zhu L., Lu Y., Wang Y., Zhang L., Wang W. (2012). Preparation and characterization of dopamine-decorated hydrophilic carbon black. Appl. Surf. Sci..

[32] Kister G., Cassanas G., Vert M., Pauvert B., Terol A. (1995). Vibrational analysis of poly(L-lactic acid). J. Raman Spectrosc..

[33] Smith P.B., Leugers A., Kang S., Hsu S.L., Yang X. (2001). An analysis of the correlation between structural anisotropy and dimensional stability for drawn poly(lactic acid) films. J. Appl. Polym. Sci..

[34] Ye W., Wang D., Zhang H., Zhou F., Liu W. (2010). Electrochemical growth of flowerlike gold nanoparticles on polydopamine modified ITO glass for SERS application. Electrochim. Acta.

[35] Yang X., Kang S., Yang Y., Aou K., Hsu S.L. (2004). Raman spectroscopic study of conformational changes in the amorphous phase of poly(lactic acid) during deformation. Polymer.

[36] Tonda-Turo C., Gentile P., Saracino S., Chiono V., Nandagiri V., Muzio G., Canuto R., Ciardelli G. (2011). Comparative analysis of gelatin scaffolds crosslinked by genipin and silane coupling agent. Int. J. Biol. Macromol..

[37] Karp J.M., Shoichet M.S., Davies J.E. (2003). Bone formation on two-dimensional poly(DL-lactide-co-glycolide) (PLGA) films and three-dimensional PLGA tissue engineering scaffoldsin vitro. J. Biomed. Mater. Res..

[38] Bhattacharya R., Das T.K., Saha S. (2011). Synthesis and characterization of CdS nanoparticles. J. Mater. Sci. Mater. Electron..

[39] Park H.H., Lee K.Y., Lee S.J., Park K.E., Park W.H. (2007). Plasma-treated poly(lactic-co-glycolic acid) nanofibers for tissue engineering. Macromol. Res..

[40] Kang S.M., You I., Cho W.K., Shon H.K., Lee T.G., Choi I.S., Karp J.M., Lee H. (2010). One-Step Modification of Superhydrophobic Surfaces by a Mussel-Inspired Polymer Coating. Angew. Chem. Int. Ed..

[41] Lee H., Rho J., Messersmith P.B. (2009). Facile Conjugation of Biomolecules onto Surfaces via Mussel Adhesive Protein Inspired Coatings. Adv. Mater..

[42] Tonda-Turo C., Carmagnola I., Ciardelli G. (2018). Quartz Crystal Microbalance with Dissipation Monitoring: A Powerful Method to Predict the in vivo Behavior of Bioengineered Surfaces. Front. Bioeng. Biotechnol..

[43] Shin Y.M., Bin Lee Y., Kim S.J., Kang J.K., Park J.-C., Jang W., Shin H. (2012). Mussel-Inspired Immobilization of Vascular Endothelial Growth Factor (VEGF) for Enhanced Endothelialization of Vascular Grafts. Biomacromolecules.

[44] Wei Q., Li B., Yi N., Su B., Yin Z., Zhang F., Li J., Zhao C. (2010). Improving the blood compatibility of material surfaces via biomolecule-immobilized mussel-inspired coatings. J. Biomed. Mater. Res. Part A.

[45] Ryou M.-H., Lee Y.M., Park J.-K., Choi J.W. (2011). Mussel-Inspired Polydopamine-Treated Polyethylene Separators for High-Power Li-Ion Batteries. Adv. Mater..

[46] Bellucci D., Sola A., Gentile P., Ciardelli G., Cannillo V. (2012). Biomimetic coating on bioactive glass-derived scaffolds mimicking bone tissue. J. Biomed. Mater. Res. Part A.

[47] Zhao S., Xie G., Huangfu X., Zhao J., Bohu Y., Klouche S., Lefevre N., Gerometta A. (2017). Gelatin-Grafted Electrospun Fibrous Membranes for Rotator Cuff Repair. Arthrosc. J. Arthrosc. Relat. Surg..

